# Investigation of the Molecular Mechanisms of Antioxidant Damage and Immune Response Downregulation in Liver of *Coilia nasus* Under Starvation Stress

**DOI:** 10.3389/fendo.2021.622315

**Published:** 2021-02-26

**Authors:** Meiyao Wang, Gangchun Xu, Yongkai Tang, Shengyan Su, Yinping Wang, Zhixiang Zhu

**Affiliations:** ^1^ Key Laboratory of Freshwater Fisheries and Germplasm Resources Utilization, Ministry of Agriculture, Freshwater Fisheries Research Center, Chinese Academy of Fishery Sciences, Wuxi, China; ^2^ Wuxi Fisheries College, Nanjing Agricultural University, Wuxi, China; ^3^ Aquatic Animal Genome Center of Freshwater Fisheries Research Center, Chinese Academy of Fishery Sciences, Wuxi, China; ^4^ Scientific Observing and Experimental Station of Fishery Resources and Environment in the Lower Reaches of the Changjiang River, Ministry of Agriculture and Rural Affairs, Wuxi, China

**Keywords:** *Coilia nasus*, liver, comparative transcriptome, starvation, immune response

## Abstract

Commercial fishing of estuarine tapertail anchovy (*Coilia nasus*), an important anadromous fish species in the Yangtze River of China, has been prohibited due to the serious damage overfishing has caused to the wild population. Research regarding the energy metabolism is important for migratory fish to ensure the continuation of their existence. In this study, we performed, for the first time, a comparative transcriptome analysis of the liver of *C. nasus* subjected to long-term starvation stress. The results indicated that the damaging effects involved downregulation of the antioxidant capacity and immune response. The positive response to starvation involved upregulation of the anti-allergy and anticancer capacity, which supports the function of starvation in cancer inhibition, as has also been determined for human beings. This study revealed regulatory pathways, differentially expressed genes (DEGs), and mechanisms leading to damage of the liver in *C. nasus* affected by starvation. This research contributes information for the further study of the energy metabolism mechanism of *C. nasus* and provides a theoretical reference for starvation metabolism research of other fish species and even human beings.

## Introduction

The Yangtze River, winding about 6,387 km, is the largest in China and the third longest river in the world. The Yangtze River is also one of seven rivers that possess abundant aquatic biodiversity. There are 4,300 aquatic organisms, including 400 kinds of fish species; 11 types of national key protection aquatic organisms, such as Chinese sturgeon (*Acipenser sinensis*), Yangtze sturgeon (Acipenser dabryanus), and Yangtze finless porpoises (*Neophocaena asiaeorientalis*); and 170 types of fish species particular to the Yangtze River (1).

*C. nasus*, which belongs to the order Clupeiformes, the family Engraulidae, and the genus *Coilia*, is an important anadromous fish species found in the Yangtze River of China. It is widely popular due to its delicate and unique flavor and is among the so-called “Three Delicious Fish Species in the Yangtze River” together with the Reeves shad (*Tenualosa reevesii*) and obscure pufferfish (*Takifugu fasciatus*) (2, 3). Under the long-term influence of water pollution, overfishing, channel improvement, water project construction, etc., the living environment of aquatic organisms in the Yangtze River is deteriorating day by day, and certain rare species populations have been diminished (4).

As an important anadromous fish species in the Yangtze River, the wild population of *C. nasus* has been seriously damaged. It has been listed as an endangered fish species in China and the Ministry of Agriculture and Rural Affairs of the People s Republic of China issued a notice prohibiting the commercial fishing of C. nasus in the Yangtze River (5). Therefore, the artificial breeding industry of *C. nasus* is burdened with the responsibility of providing high-quality fish fry for reproduction and release to restore the wild resources of *C. nasus* in the Yangtze River while also continuing to enrich people’s dining tables.

Adult individuals of *C. nasus*, as an anadromous fish in the Yangtze River, undergo reproductive migration along the Yangtze River every February when their gonads have matured. Juvenile *C. nasus* will settle in the estuarine area until autumn and then undergo seaward migration for growth and fattening (6, 7). During the migration process, starvation is most likely to be caused by climate change, regional change, and uneven food distribution.

Therefore, the regulation of the robust energy metabolism mechanism of *C. nasus* during its migration is an important topic of research. In addition, in the daily artificial breeding of *C. nasus*, fish can face starvation due to excessive stocking density, delayed feeding, and uneven feeding. As a result, it is necessary to explore the metabolic mechanisms of *C. nasus* under starvation stress, which is of positive significance for revealing the regulatory mechanism of the energy metabolism in this species with additional benefits for the restoration of its wild resources in the Yangtze River as well as development of the artificial breeding industry.

The liver is a key organ in fish energy metabolisms (8). At present, there are several reports on the influence of starvation on the livers of fish, including yellow drum (*Nibea albiflora*) (9), large yellow croaker (*Pseudosciaena crocea*) (10), red sea bream (*Pagrus major*) (11), zebrafish (*Danio rerio*) (12, 13), and common dentex (*Dentex dentex*) (14). Authors in these studies examined the effects of cold and starvation on the mode of fish metabolism and discussed the metabolic mechanisms of starvation or starvation/refeeding as it relates to liver physiology, nutrient metabolism, and energy-related pathways, such as for the fatty acid metabolism, antioxidant responses, and the immune responses of fish.

The results indicated that crude fat content can be used as a biomarker for the starvation response. In addition, although starvation caused body oxidative stress in the short term, long-term starvation resulted in the failure of cellular antioxidant defenses. Fish have been found to utilize noncarbohydrate substances to produce energy under starvation stress, which causes the enhancement of gluconeogenesis (9–14). On the other hand, comparative transcriptome analyses of fish livers have been carried out to explore the regulatory mechanism of the liver under starvation stress at a broader scope.

Relevant studies have been carried out on large yellow croaker (*Larimichthys crocea*) (15), Atlantic salmon (*Salmo salar*) (16), zebrafish (*Danio rerio*) (17), and rainbow trout (*Oncorhynchus mykiss*) (18). The effects of starvation or starvation/refeeding on the energy metabolism and the immune response in fish livers were investigated. The most significant involved pathways were mainly those of fat digestion and absorption, the citrate cycle, and glycolysis/gluconeogenesis. There have been few reports of differential pathways relevant to the immune response: in this category, only coagulation cascades were identified (15). Starvation caused the downregulation of many immune-related pathways and regulatory genes in Atlantic salmon (16).

Research on zebrafish indicates that starvation causes significant downregulation of the metabolic activity, including lipid metabolism, protein biosynthesis, proteolysis, and cellular respiration, but results in the upregulation of gluconeogenesis (17). Research on rainbow trout revealed that when fish weight was significantly reduced due to starvation, calpain and 20S proteasome pathways in protein mobilization functioned as an energy source (18). The above studies have shown that, under starvation conditions, the livers of different fish species undergo differential regulation modes.

There have been few reports on the regulatory mechanism of *C. nasus* under starvation stress. In one of the only studies on this subject, Jin Xin et al. discussed the effect of starvation stress on the chemical composition and blood biochemical parameters of *C. nasus* (19). Therefore, we conducted the first comparative transcriptome analysis of the liver of near-death *C. nasus* under long-term starvation stress to reveal key regulatory pathways and genes. We aimed to reveal the mechanisms leading to damage of the liver in *C. nasus* under starvation stress as well as to lay a theoretical foundation for the investigation of the comprehensive energy metabolism mechanisms of *C. nasus* providing a theoretical reference for further in-depth research on the restoration of wild *C. nasus* resources and for the artificial breeding industry.

## Materials and Methods

### Ethical Statement

This study was approved by the Animal Care and Use Committee of the Freshwater Fisheries Research Center at the Chinese Academy of Fishery Sciences. The experiments were carried out in accordance with the Guidelines for the Care and Use of Laboratory Animals set by the Animal Care and Use Committee of the Freshwater Fisheries Research Center (2003WXEP61, Jan 6th of 2003), and the study was carried out with a field permit (no. 20176CN1619).

### Experimental Fish and Starvation Stress

Experimental fish were 5-month-old juvenile *C. nasus* with an average body weight of 8.6 ± 0.59 g and a body length of 143.6 ± 0.82 mm. The fish were collected from Yixing, an experimental base of the Freshwater Fisheries Research Center of the Chinese Academy of Fishery Sciences. One month before the starvation experiment, juvenile *C. nasus* were netted and cultured in four aquariums. The aquariums were equipped with circulating water systems. The water quality was measured once each day, the pH was at 7.1, and water temperature was 18 ± 0.62°C. The concentration of dissolved oxygen was 7.8 ± 0.65 mg/L. The experimental fish were fed three times per day.

A control group (C) and a starvation group (S) were established. Each group had two replicates and each aquarium contained ten fish. The stressed group was not fed during the experimental period. On the 26th day of the starvation experiment, we observed that some fish in the stressed group swam laterally and were near death; these fish were caught, placed in 40 mg/L MS-222 solution (Kuer Bioengineering, Beijing, China) for rapid anesthesia and then dissected. We observed that the muscles and internal organs of the sampled fish had atrophied. Then, the remaining excessive starvation fish were also quickly caught and anesthetized. Two fish were sampled from each aquarium. The livers were collected and snap-frozen in liquid nitrogen and then stored at −80°C until further treatment.

### Total RNA Extraction, cDNA Library Construction, and Illumina Sequencing

The total RNA for each sample was extracted using TRIzol reagent (Invitrogen) following the manufacturer’s instructions. Equal amounts of RNA from fish in the same tank were pooled as one sample to obtain a total of four RNA samples. The qualification and quantification of RNA, cDNA library construction, and Illumina sequencing, were subsequently carried out according to methods in our previously reported study (20). The obtained raw data were submitted to NCBI (NCBI, Bethesda, USA) with accession number PRJNA359698.

### Data Filtering and Assembly

Quality control for the raw data was carried out using FASTQC (Babraham Institute, Cambridge, UK). Some primers, adapters, low-quality reads, etc., were processed by Cutadapt (version 1.9.1) (21). The assembly of the transcriptome data was carried out using Trinity (version 2.4.0) with min_kmer_cov set to 2 by default and all other parameters set to default (22).

### Function Annotation

Unigenes were aligned on the basis of nonredundant protein (Nr), nonredundant nucleotide (Nt), Swiss-Prot (http://www.uniprot.org/downloads), clusters of orthologous groups for complete eukaryotic genomes (COG, ftp://ftp.ncbi.nih.gov/pub/COG/KOG/kyva), Gene Ontology (GO, http://www.geneontology.org/), and the Kyoto Encyclopedia of Genes and Genomes (KEGG, http://www.genome.jp/kegg/pathway.html) using BlastX (version 2.2.28+) with an E-value <10^–5^ (23, 24). GO annotation was performed using Blast2GO (version b2g4pipe_v2.5) (Biobam, Valencia, Spain) (25).

### Gene Quantification and Differential Expression Analysis

The assembled unigenes were placed in a constructed library, and the abundance of the expression of each unigene in each sample was measured using Bowtie2 (version 2.1.0) (http://bowtie-bio.sourceforge.net/bowtie2/manual.shtml) (Ben Langmead, Maryland, College Park, USA) (26) and eXpress software (version 1.5.1) (http://www.rna-seqblog.com/express-a-tool-for-quantification-of-rna-seq-data/) (California University, Berkeley, USA) (27). For the software parameters in Bowtie, -k was set to 30, and -t was the default value. In the eXpress software, the strand-specific mode was set to –rf-stranded. The gene expression levels were evaluated as fragments per kilobase of transcript per million mapped reads (FPKM) (28). Differential expression analysis was performed using the DESeq software R package (version no. 1.18.0) (29) (http://bioconductor.org/packages/release/bioc/html/DESeq.html). The resulting *p*-values were adjusted using Benjamini and Hochberg’s approach to control the false discovery rate. The fold change in gene expression was calculated as the ratio between the expression level of genes in the starvation stress group and in the control group. Genes with an adjusted *p*-value (padj) < 0.05 and |log_2_foldchange| > 1 found by DESeq were considered as being differentially expressed. Gene Ontology (GO) enrichment analysis of differentially expressed genes (DEGs) was implemented in the GOseq R packages based on Wallenius’ noncentral hypergeometric distribution (30). The GO terms enriched from DEGs were sequenced with −log_10_ (*p*-value), and the top 30 GO terms (including three subcategories, which were biological process (BP), cellular component (CC), and molecular function (MF)) were filtered according to −log_10_ (*p*-value) and ordered according to DEG numbers. Finally, we obtained the top 30 GO terms. We used KOBAS software to test the statistical enrichment of differentially expressed genes in the Kyoto Encyclopedia of Genes and Genomes (KEGG) pathways (31). The KEGG terms were sequenced with the −log_10_ (*p*-value), and the top 20 KEGG pathways were filtered according to the −log_10_ (*p*-value) and ordered according to the DEG numbers. Finally, we obtained the top 10 KEGG pathways. The above transcriptome data analysis, including function annotation, gene quantification, and differential expression analysis, was carried out following the method reported in our previous study (20).

### Quantitative Real-time PCR Validation

To validate the accuracy of high-throughput sequencing results, ten DEGs were randomly selected from the transcriptome data for qRT-PCR analysis. The genes and primers sequences were shown in **Supplementary file S1**. The qRT-PCR validation experiment was carried out on the ABI 7500 real-time PCR system (ABI, New York, USA). The primers were designed using Primer Premier 5 software (Premier Biosoft, California, USA) (32), and the sequences of the designed primers are shown in **Appendix S1**. Beta-actin was used as the internal reference. The amplifications were conducted in a 10 μl reactions, which contained 2 ng of total RNA, 5 μl master mixes, and 0.4 μl of each primer. The qRT-PCR reactions were conducted with the following procedure: 95°C for 30 s, then 40 cycles of 95°C for 5 s, 60°C for 34 s, and then 72°C for 50 s. All samples were assayed in triplicate, and the 2^−ΔΔCT^ method was used to calculate the expression level of the ten DEGs. Cycle threshold (C_T)_ refers to the PCR cycle number at which the fluorescence signal crossed a threshold line that was placed in the exponential phase of the amplification curve. ΔΔC_T_ = (C_T.S_ – C_T.actin_) – (C_T.C_ – C_T.actin_) where C_T.S_ refers to the C_T_ value of samples in the starvation group, C_T.C_ refers to the C_T_ value of samples in the control group (33).

### Statistical Analysis

Statistical analyses were carried out with SPSS 21.1 software (SPSS, Chicago, USA). The values are shown as the means ± SE. Statistical analysis was performed with Student’s t-test, and *p* < 0.05 was set as the significant difference standard.

## Results

### Statistics Analysis of Transcriptome Data in the Liver of *C. nasus* Under Starvation Stress

The statistics of high-throughput sequencing data are shown in **Table 1**. Q30 refers to the proportion of bases with a base error rate of 0.1%. In this study, the Q30 of each group was higher than 95%, indicating that the transcriptome data had high accuracy.

**Table 1:** Statistics of transcriptome data of liver in *C. nasus* under starvation.

Samples	Raw reads	Clean reads	Q30(%)	Unigenes	≥500 bp unigenes	≥1,000 bp unigenes	N50 length (bp)
C1	46,289,510	46,288,119	95.3	211,229	136,126	50,319	1,466
C2	46,266,980	46,265,236	95.1				
S1	46,528,969	46,527,116	96.8				
S2	46,514,062	46,513,086	95.3				

### Top 30 GO Terms Enriched From DEGs

**Figure 1:**
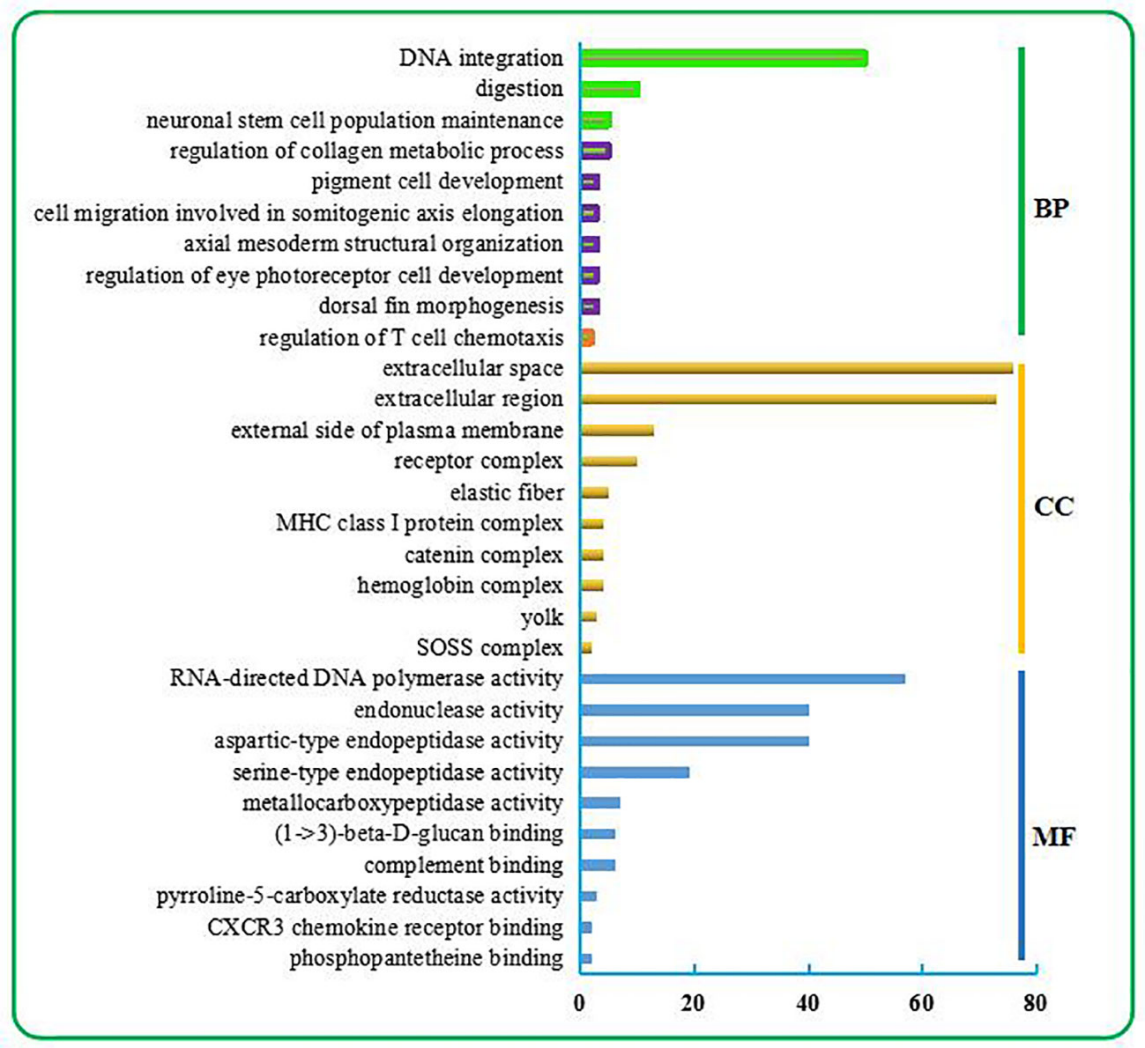
Top 30 GO terms enriched from the liver transcriptome of *C. nasus* under starvation stress. The top 30 GO terms include the top 10 terms in BP, CC, and MF categories. BP terms were further classified. Three terms relevant to metabolism are marked in the green box. Six terms relevant to development are marked in the purple box. One term relevant to the immune response is marked in the orange box.

As shown in **Figure 1**, the top 10 BP terms were mainly involved in the regulation of metabolism, development, and the stress response. Among these were the metabolism involved in the nucleic acid metabolic process (no. 1) and regulation of metabolic process (nos. 2–3); developmental regulation through maintenance of cell number (no. 4), regulation of cell migration (no. 8), cell development (no. 9), tissue morphogenesis (no. 5), tissue structure arrangement regulation (no. 7), regulation of neuron differentiation (no. 6); and stress response regulation, which was mainly related to the immune response (no. 10).

### Top 10 KEGG Pathways

**Figure 2:**
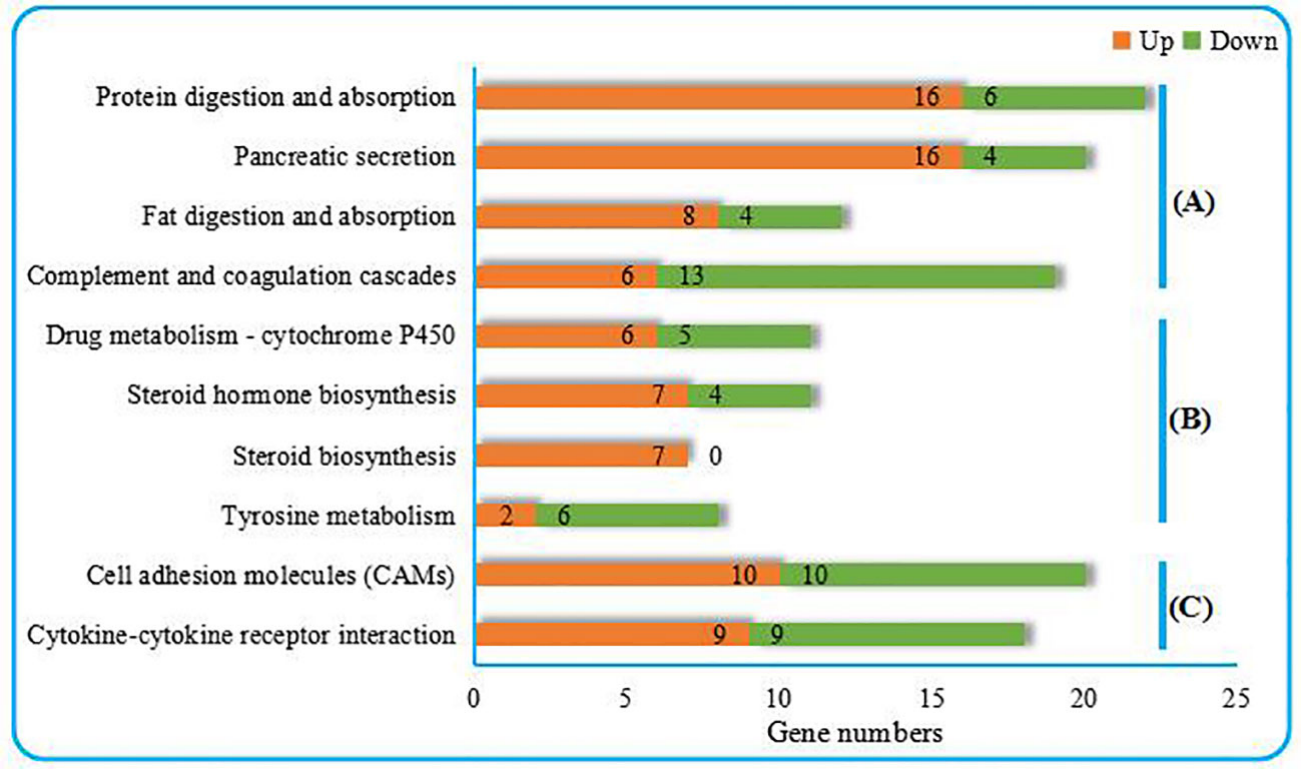
Top 10 KEGG pathways enriched from the liver transcriptome of *C. nasus* under starvation stress. **(A)** Organismal systems, **(B)** Metabolism, **(C)** Environmental information processing. Numbers marked by orange bars and green bars indicate the numbers of upregulated and downregulated DEGs.

As shown in **Figure 2**, the top 10 KEGG pathways were related to the digestion metabolism (nos. 1–3, 5–8) and mainly involved the energy-substance metabolism, including lipids, proteins, and amino acids. In addition, the top 10 KEGG pathways also involved signal transduction regulation, which was mainly related to immune response regulation (nos. 4, 9–10).

### Functional Analysis of Key DEGs

The key DEGs in the top 10 BP and top 10 KEGG pathways were comprehensively analyzed, and they were found to mainly involve the energy-substance metabolism and immune response regulation (**Table 2**).

**Table 2:** Key DEGs in top 10 BP and top 10 KEGG.

Category	Gene name	Definition	log_2_Foldchange	padj
Energy substance metabolism	CEL	Bile salt-activated lipase	4.20	7.40E-08
	ABCG1	ATP-binding cassette sub-family G member 1	1.63	2.35E-04
	ABCG5	ATP-binding cassette sub-family G member 5	1.70	2.89E-04
	DGAT1	Diacylglycerol O-acyltransferase 1	2.64	4.91E-05
	UGT1A5	UDP-glucuronosyltransferase 1–5	3.46	6.59E-05
	UGT2A1	UDP-glucuronosyltransferase 2A1	3.42	6.66E-07
	UGT2B31	UDP-glucuronosyltransferase 2B31	2.34	3.93E-05
	UGT2C1	UDP-glucuronosyltransferase 2C1	1.96	1.30E-04
	COMT	Catechol O-methyltransferase	2.29	8.47E-05
	CYP7A1	Cholesterol 7-alpha-monooxygenase	1.87	2.54E-04
	EBP	3-beta-hydroxysteroid-Delta(8),Delta(7)-isomerase	2.00	1.46E-04
	DHCR7	7-dehydrocholesterol reductase	2.00	1.18E-04
	LSS	Lanosterol synthase	2.19	6.13E-05
	LIPA	Lysosomal acid lipase/cholesteryl ester hydrolase	1.87	1.67E-04
	SQLE	Squalene monooxygenase	1.99	1.16E-04
	HSD11B2	Corticosteroid 11-beta-dehydrogenase isozyme 2	−4.03	1.90E-04
	ALDH1A3	Aldehyde dehydrogenase family 1 member A3	−2.49	1.90E-04
	ALDH3B1	Aldehyde dehydrogenase family 3 member B1	−2.38	3.44E-05
	HPD	4-hydroxyphenylpyruvate dioxygenase	−2.55	1.70E-05
Immune response regulation	BDKRB2	B2 bradykinin receptor	−6.07	1.35E-05
	CLU	Clusterin	2.84	1.99E-04
	CDH2	Cadherin-2	3.24	2.40E-05
	LRRC4C	Leucine-rich repeat-containing protein 4C	2.30	2.23E-04
	HGF	Hepatocyte growth factor	−1.69	2.61E-04
	MET	Hepatocyte growth factor receptor	−2.57	4.25E-05
	CD276	CD276 antigen	2.42	3.96E-05
	CNTN1A	Contactin-1a	−1.73	3.07E-04
	C3	Complement C3	−5.26	2.59E-06
	C6	Complement component C6	−2.48	3.08E-05
	ITGAM	Integrin alpha-M	−2.57	3.59E-04
	ITGB2	Integrin beta-2	−2.67	1.40E-05
	ITGA6	Integrin alpha-6	−2.26	3.43E-04
	VWF	von Willebrand factor	−2.89	9.38E-05
	CD22	B-cell receptor CD22	–	1.40E-04
	A2ML1	Alpha-2-macroglobulin-like protein 1α2	−2.89	8.88E-06
	IL6ST	Interleukin-6 receptor subunit beta	3.84	2.99E-04
	CXCL12	Stromal cell-derived factor 1	−2.86	5.68E-05
	TNFRSF14	Tumor necrosis factor receptor superfamily member 14	+	4.34E-06

‘-’ indicates the DEG was not expressed in the stressed group; ‘+’ indicates that the DEG was not expressed in the control group.

### Validation of RNA-Seq Data by qPCR

**Figure 3:**
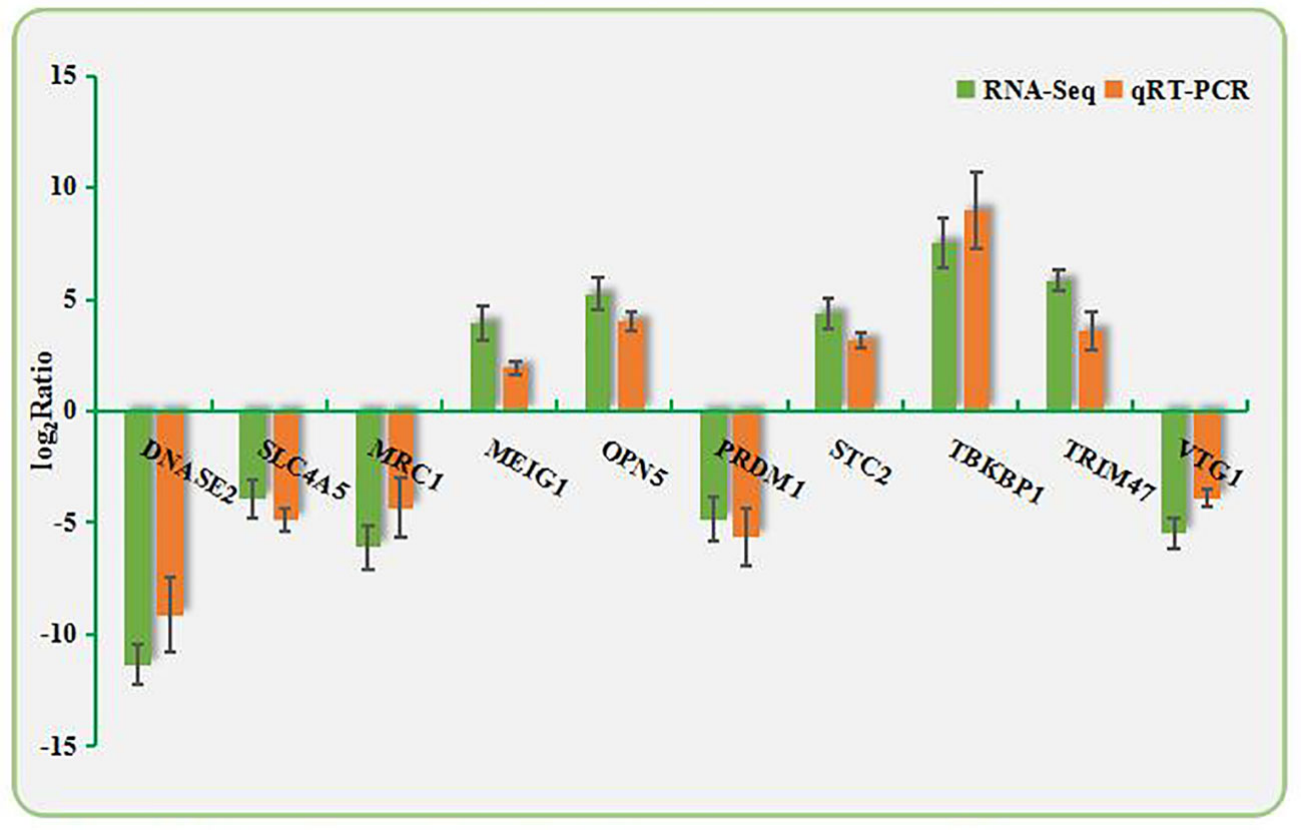
Validation of RNA-Seq data by qPCR. The X-axis shows the names of the assayed gene; the Y-axis is the relative expression level, expressed as log2 (fold change) in gene expression.

Ten DEGs were randomly selected from the RNA-Seq data. qPCR was performed using the primers shown in **Appendix A1**. As shown in **Figure 3**, the directions of changes in the detected DEGs were concordant between the RNA-Seq and qPCR analyses.

## Discussion

The top 10 BPs, top 10 KEGG pathways, and the related DEGs were comprehensively analyzed, and the results show that they are mainly involved in the energy-substance metabolism and immune response regulation. The results related to the energy-substance metabolism mainly involved lipid catabolism and transportation, hormone regulation, and antioxidant regulation, and those involving the immune response were mainly related to immune response regulation and antitumor regulation.

### Energy-Substance Metabolism

#### Enhancement of Lipid Catabolism

In this study, many DEGs related to lipid catabolism were upregulated after starvation stress, including bile salt-activated lipase (*CEL*), ATP-binding cassette subfamily G member 1 (*ABCG1*), and ATP-binding cassette subfamily G member 5 (*ABCG5*). *CEL*, a lipolytic enzyme synthesized and secreted by the pancreas, is conducive to the decomposition and digestion of fat and also participates in the lipoprotein metabolism. CEL can also promote the formation of capillary networks, cell proliferation, cell migration, tissue damage repair, etc. (34, 35).

In this study, *CEL* was upregulated after starvation stress, thereby, increasing the energy supply and maintaining the energy metabolism balance. *ABCG1*, which catalyzes the efflux of phospholipids, such as sphingolipids and cholesterol, participates in intracellular lipid transportation (36). *ABCG5*, which mediates the transport of Mg^2+^ and ATP-dependent sterols through the cell membrane, plays an important role in the selective transport of cholesterol into and out of intestinal epithelial cells (37). The upregulation of these DEGs promotes the catabolism of fat and lipid transport for energy supply during the starvation period.

The research performed by Lin et al. (38) indicated that feed restriction caused upregulation of the lipid catabolism in the liver of green sturgeon (*Acipenser medirostris*), and they obtained similar results to this study. The research carried out by Escalante-Rojas et al. (39) showed downregulation of the lipid reserves in the liver of rose spotted snappers (*Lutjanus guttatus*) under starvation conditions. The research performed by Qian et al. (15) also indicated that many regulatory genes related to lipid catabolism in the liver of large yellow croakers (*Larimichthys crocea*) were upregulated, which is beneficial for the reallocation of lipid reserves.

#### Enhancement of Detoxication

In this study, some DEGs relevant to detoxification were significantly upregulated in the liver of *C. nasus* after starvation stress, including diacylglycerol o-acyltransferase 1 (*DGAT1*), UDP-glucuronosyltransferase 1A5 (*UGT1A5*), UDP-glucuronosyltransferase 2A1 (*UGT2A1*), UDP-glucuronosyltransferase 2B31 (*UGT2B31*), and UDP-glucuronosyltransferase 2C1 (*UGT2C1*). *DGAT1* plays an important regulatory role in maintaining the homeostasis of retinol (namely, vitamin A) and preventing retinol poisoning (40). UDP-glucuronide transferase (*UGT*) can catalyze the binding of lipophilic substrates with glucuronic acid to increase the water solubility of metabolites, thus, promoting excretion into the urine or bile. *UGT* is essential for the elimination and detoxification of drugs, invading organisms, and endogenous compounds (41, 42). In this study, many UGTs, such as *UGT1A5*, *UGT2A1*, *UGT2B31*, and *UGT2C1*, were upregulated after starvation. They play an important role in binding and subsequently removing potentially toxic exogenous and endogenous compounds. The research conducted by Xu et al. (43) demonstrated that starvation can lead to the upregulation of UDP-glucuronosyltransferase in the liver of mice, which plays an important role in modulating the hormones level and the elimination of harmful substances.

#### Enhancement of Hormone Regulation

Here, some hormone regulation-relevant DEGs were upregulated, such as catechol o-methyltransferase (*COMT*), cholesterol 7-alpha-monooxygenase (*CYP7A1*), 3-beta-hydroxysteroid-delta (8) delta(7)-isomerase (*EBP*), 7-dehydrocholesterol reductase (DHCR7), lanosterol synthase (*LSS*), lysosomal acid lipase/cholesteryl ester hydrolase (*LIPA*), and squalene monooxygenase (*SQLE*). *COMT* is beneficial for inactivating the neurotransmitter function of catecholamine, thus, slowing down the metabolism and conserving energy (44).

*COMT* upregulation is beneficial for the maintenance of energy homeostasis during the starvation period. *CYP7A1* is a rate-determining enzyme for bile acid synthesis that is exclusively expressed in the liver, and it can increase the hydrolysis of lipids. Bile acid can save energy and improve its utilization (45). According to this study, its upregulation may be beneficial for energy homeostasis maintenance during starvation stress.

*EBP*, *DHCR7*, and LSS are key enzymes in the cholesterol biosynthesis pathway (46–48). *LIPA* can catalyze the deacylation of the cholesterol core ester to generate free fatty acids and cholesterol (49). *SQLE* is a rate-limiting enzyme in steride biosynthesis and an important enzyme for catalyzing the formation of cholesterol precursor molecules (50). Free fatty acids and cholesterol are also important energy-supplying substances. According to this study, the upregulation of these DEGs may enhance the energy supply of *C. nasus* during starvation stress.

#### Weakening of Water and Salt Metabolism and Antioxidation

Corticosteroid 11-beta-dehydrogenase isozyme 2 (*HSD11B2*) can catalyze the conversion of cortisol to inactive cortisone and protect nonselective mineralocorticoid receptors from glucocorticoid occupation. *HSD11B2* plays a direct regulatory role in the water and salt metabolism in the kidney and skin to maintain blood pressure balance (51). To some extent, its downregulation reflects the downregulation of water and salt metabolism induced by starvation in this study. The results of the research carried out by Lin et al. (38) indicated that restricted feeding caused downregulation of the osmolality in the liver of green sturgeons (*Acipenser medirostris*).

Acetaldehyde dehydrogenase is the most important enzymatic aldehyde metabolism system in cells. It plays an important role in the substance metabolism, embryo formation, cell proliferation, and differentiation (52). In the presence of NAD(P)^+^, aldehyde dehydrogenase family 1 member A3 (*ALDH1A3*) oxidizes acetaldehyde to acetic acid, thereby, participating in the detoxification of aldehydes (53, 54). Aldehyde dehydrogenase family of 3 member B1 (*ALDH3B1*) can oxidize medium- and long-chain saturated and unsaturated aldehydes and has a strong antioxidant function, allowing it to protect against cytotoxicity caused by lipid peroxidation (55).

Vitamin E, also known as tocopherol, has strong antioxidant functions and plays an important regulatory role in intracellular signal transduction (56). The necessary enzyme for the first step of tocopherol synthesis is 4-hydroxyphenylpyruvate dioxygenase (*HPD*) (57). Here, the downregulation of the above DEGs hindered the synthesis of vitamin E and other antioxidants and weakened the antioxidant capacity of *C. nasus*.

A previous study on the liver of zebrafish under starvation stress indicated that starvation caused weakening of the antioxidant capacity of the liver, and *ALDH3B1* and *HPD* were also downregulated (17). Similar conclusions were also obtained in a study on the influence of starvation on the liver of rainbow trout (18), which reflects the significant effect of starvation on the antioxidant system of fish. The research conducted by Varju et al. (58) indicated that excessive starvation caused an impaired antioxidant response in liver of pikeperch (*Sander lucioperca L.*), and they drew similar conclusions to this research.

### Regulation of the Immune Response

#### Improvement in Anti-Allergy and Anticancer Regulation

Bradykinin can expand blood vessels, increase blood vessel permeability, induce bronchospasm, and promote bronchial contraction and other allergic reactions by binding to bradykinin receptors (*BDKRB2*) on the surfaces of target cells (59). In this study, bradykinin downregulation was beneficial for decreasing the production of allergic reactions, which is of positive significance to *C. nasus*. Clusterin (*CLU*), as an extracellular partner, can block the aggregation of nonnatural proteins, stabilize mitochondrial membrane integrity, and thus inhibit stress-induced apoptosis.

*CLU* plays an important role in the clearance of immunocomplexes during cell damage (60). In this study, its upregulation was beneficial to the timely clearance of immune complexes after cell injury. Cadherin-2 (*CDH2*), a calcian-dependent cellular adhesion protein, preferentially mediates intercellular adhesion of homotypic cells, inhibits cell migration, and thus effectively inhibits tumor invasion and metastasis (61). Antitumor studies on human beings (*Homo sapiens*) demonstrated that starvation may be an effective method in tumor treatment (62, 63). This study also derived the same conclusion.

Leucine-rich repeat-containing protein 4C (*LRRC4C*) is involved in the inhibition of glioma, induction of the antitumor immune response, and activation of natural killer cells in the destruction of tumor cells (64). Here, *LRRC4C* upregulation indicated an enhanced antitumor ability (65). On the other hand, many DEGs related to tumor development, such as hepatocyte growth factor (*HGF*) and hepatocyte growth factor receptor (MET), were downregulated. Upon binding to its receptor MET, HGF can promote the invasion and metastasis of tumor cells (66). In this study, the downregulation of these DEGs implies the weakening of carcinogenicity.

#### Weakening of the Immune Response

Here, the regulation of the immune response in *C. nasus* after starvation was weakened. Complement 3 (*C3*) plays a central role in the activation of the complement system. *C3* plays an important role both in classical and bypass activation pathways of complement (67). Complement 6 (*C6*) plays an important role in cytolysis, the inflammatory response, and immune regulation (68). In a previous study on rainbow trout, C3 was also significantly downregulated after starvation stress, which is consistent with the results of this study (18). Together, these findings demonstrate that starvation can cause the weakening of complement function and, thereby, weaken the immune responses of fish.

In addition, we found some lectins to be downregulated after starvation stress. Integrin alpha-M (*ITGAM*) participates in various adhesion interactions among monocytes, macrophages, and granulocytes and mediates the uptake of pathogens. It shares a fragment with the C3 receptor and can interact with C3 to jointly mediate the immune response (69). Integrin beta-2 (*ITGB2*), contributing to natural killer cytotoxicity, participates in leukocyte adhesion and transport, including T cells and neutrophils, and mediates the immune response (70). Integrin alpha-6 (*ITGA6*), which is a receptor for platelet laminin, plays an important regulatory role in the signal transduction of insulin-like growth factor.

Insulin-like growth factor plays an important regulatory role in the proliferation and function of thymus and immune-competent cells (71). Therefore, *ITGA6* also plays an important regulatory role in the immune response. Von Willebrand factor (*VWF*) plays an important role in hemostasis. *VWF* is a partner of coagulation factor VIII and can deliver it to the site of injury, stabilize its heterodimeric structure, and protect it from being removed prematurely (72).

Here, its downregulation indicated, in a sense, a decline in coagulation ability. B-cell receptor CD22 (*CD22*) can mediate the interaction between B cells, participate in the localization of B cells in lymphoid tissues, and play an important regulatory role in signal transduction between antigen and receptor during the immune response (73). Research on the influence of starvation on zebrafish liver also indicated the downregulation of *CD22* (17), which is consistent with the results of this study.

Alpha-2-macroglobulin-like protein 1 (*A2ML1*) affects the activity of proteolytic enzymes, such as chymotrypsin and papain, through direct binding. As a result, the activity of certain proteases can be selectively protected to maintain homeostasis, which is also beneficial for the immune response (74). In this study, its downregulation weakened the protective effect on proteolytic enzymes and was not conducive to the maintenance of homeostasis.

Interleukin-6 receptor subunit beta (*IL6ST*), which is secreted by macrophages and contributes to cytokine effects, can stimulate and participate in the proliferation and differentiation of immune cells. *IL6ST* also affects the release of cortisol and plays a regulatory role in homeostasis (75). In this study, its downregulation was associated with a decrease in homeostasis regulation. Stromal cell-derived factor 1 (*CXCL12*), as a chemokine, performs a regulatory role in the chemotaxis of T cells and monocytes in the immune response (76).

Here, its downregulation reflected a decline in the regulation of the immune response. Tumor necrosis factor receptor superfamily member 14 (*TNFRSF14*), as a receptor of TNF superfamily members, as well as lymphotoxin and immunoglobulin superfamily members can promote the survival and differentiation of immune cells and the proliferation of T cells. TNFRSF14 plays a leading role in the adaptive immune response and provides a survival signal to effector T cells (77). In this study, its downregulation in the liver of *C. nasus* after starvation is consistent with the results obtained in previous research on the effect of starvation on the liver of Atlantic salmon (16).

### This Study and the Influence of Environmental Pollution and Climate Change on Wild Fish and Aquaculture

Environmental pollution and climate change have had adverse effects on the ecological environment of natural waters including oceans, lakes, and rivers. Climate change effects, such as El Nino, have resulted in abnormal temperature rises, substantial reductions in plankton, mass migrations of fish, and changed distributions of fish. New pathogens are brought to indigenous fish with the invasion of fish. In addition, temperature rises cause a lack of food, which affects the growth of fish, resulting in a decline in fertility and even death (78, 79).

The results of this study indicated that lipid catabolism was upregulated in the liver of *C. nasus* under starvation, and the antioxidant capability and immunity were downregulated. These adverse physiological alterations in fish were consistent with the unfavorable effects of climate change that lead to declines of the fish population in natural waters and decreases in the aquatic biodiversity. Similarly, climate change also affects the ecological environment of aquaculture ponds and influences the growth, survival, and reproduction of fish species (79). Therefore, to better cope with climate change, marine protection areas should be established to control pollution and overfishing, which will be beneficial for the restoration of the fish population and enhancement of resilience of the waters to climate change.

Water corridors can be established in the Yangtze River, and conservation areas can be networked to help fish find suitable habitats more safely under variable water temperatures and chemical environments. On the other hand, scientific research on selective breeding should be further intensified. Individuals with good characteristics, including fast growth and strong resistance (strong resistance to diseases and extreme weather), should be selected out. Transgenic techniques can be further improved and utilized to obtain genetically improved new varieties and, finally, to improve the germplasm of aquatic organisms in natural waters and aquaculture ponds to better cope with environmental pollution and climate change.

## Conclusions

We performed a comparative transcriptome analysis of the livers of near-death *C. nasus* after long-term starvation stress. The results showed that the main damaging effects involved the decreased regulation of water and salt metabolism, decreased antioxidant capacity, and the weakening of the immune response. The positive responses in *C. nasus* were the upregulation of certain detoxification regulatory genes and, more importantly, the upregulation of anti-allergy and anti-cancer regulatory genes as well as the downregulation of DEGs related to tumor development.

This research on *C. nasus* supports the important function of starvation in cancer inhibition. The results of this study revealed key regulatory pathways, DEGs, and the mechanisms leading to damage of the liver in *C. nasus* caused by starvation, which contributes essential information for further study of the energy metabolism mechanism of *C. nasus* as well as provides a theoretical reference for starvation metabolism research in other fish species and even human beings.

## Data Availability Statement

The data sets presented in this study can be found in online repositories. The names of the repository/repositories and accession number(s) can be found in the article/**Supplementary Material**.

## Ethics Statement

The animal study was reviewed and approved by the Animal Care and Use Committee of the Freshwater Fisheries Research Center at the Chinese Academy of Fishery Sciences.

## Author Contributions

GX supervised the research. MW designed, performed experiments, analyzed the transcriptomic data, and wrote the manuscript. ZZ and YT assisted the sampling. SS and YW made contritions to the revised manuscript. All authors contributed to the article and approved the submitted version.

## Funding

This research was funded by the National Key R & D Program of China (2019YFD0901203), the National Natural Science Foundations of China (31672643), the Central Public-Interest Scientific Institution Basal Research Fund, Freshwater Fisheries Research Center, CAFS (2018JBFR03), and the National Infrastructure of Fishery Germplasm Resources (2017DKA3047-003).

## Conflict of Interest

The authors declare that the research was conducted in the absence of any commercial or financial relationships that could be construed as a potential conflict of interest.

## Supplementary Material

The Supplementary Material for this article online at: https://www.frontiersin.org/articles/10.3389/fendo.2021.622315/full#supplementary-material

## Abbreviations

*C. nasus, Coilia nasus*; KOG, clusters of orthologous groups for eukaryotic complete genomes; GO, gene ontology; KEGG, Kyoto Encyclopedia of Genes and Genomes; DEGs, differentially expressed genes; BP, Biological process; CC, Cellular component; MF, Molecular function; qPCR, quantitative real-time polymerase chain reaction; C_T_, cycle threshold.
