# Supplementary material for: Investigation of the Molecular Mechanisms of Antioxidant Damage and Immune Response Downregulation in Liver of Coilia nasus Under Starvation Stress
Source: Front Endocrinol (Lausanne). 2021 Feb 26;12:622315. doi: 10.3389/fendo.2021.622315 (PMC7959721; doi:10.3389/fendo.2021.622315)
Supplement: Supplementary file 1 [file Table_1.docx]

**Table S1. The genes and primers used for Real-time RT-PCR validation**

| Number | Gene name | Gene definition | Sequence(5’→3’) |
| --- | --- | --- | --- |
| 1 | DNASE2 | Deoxyribonuclease-2-alpha | F: CGTTTGTTGGGGTACTGCAC |
|  |  |  | R: GGTGCATGTATGCCACTCCT |
| 2 | SLC4A5 | Electrogenic sodium bicarbonate cotransporter 4 | F: TCATGGACCAGCAGATCACG |
|  |  |  | R: CACCGGCATGGGAATGTACT |
| 3 | MRC1 | Macrophage mannose receptor 1 | F: AGGGATGAAGTCTTTCTTCGTTG |
|  |  |  | R: GATAGAAATAGAGCATCGTCGAG |
| 4 | MEIG1 | Meiosis expressed gene 1 protein homolog | F: CCAGACTCATCCCCAGATACC |
|  |  |  | R: CGATGGGATAGGGCATGTCG |
| 5 | OPN5 | Opsin-5 | F: GCAGGCTTTTGTTCTCAGCAT |
|  |  |  | R: CACAGATCAGCATTGCCACC |
| 6 | PRDM1 | PR domain zinc finger protein 1 | F: CCATCGAGTCTTTGAACGCA |
|  |  |  | R: GCATGTCGGTACGCTCCAG |
| 7 | STC2 | Stanniocalcin-2 | F: GCTGGGCCAGTTCATGACC |
|  |  |  | R: CCACACCCCACATCGCCAG |
| 8 | TBKBP1 | TANK-binding kinase 1-binding protein 1 | F: GCTCTCTCTCTGTCGAGACC |
|  |  |  | R: GTGAACACGGCACCAATCAC |
| 9 | TRIM47 | Tripartite motif-containing protein 47 | F: AGGAGATCCTCAATCACGTCG |
|  |  |  | R: CTGGATGTCCTCGATACGGAC |
| 10 | VTG1 | Vitellogenin | F: GTGACGTTTCTGCCCCAGAT |
|  |  |  | R: CAACTGTGCAGCAGTATCAGC |
| 11 | β-actin | beta-actin | F: GCAACACGCAGCTCGTTGTAG |
|  |  |  | R: CAGGCATCAGGGTGTGATGG |
